# 

**Published:** 1871-06

**Authors:** 


### CONTENTS:

ORIGINAL CONTRIBUTIONS.

Specific Action of Tissues, and their Elements as connected with the Living Body. By Daniel Lichty, M.D......... 325

The Legal Principles involved in Official Efforts for the Regulation or Suppression of Prostitution. By George Andrews, Esq., Attorney at Law; late Judge of the Supreme Court of Tennesee....... 334

Carbolic Acid in Vascular Keratitis. By C. Hixon, M.D.. formerly Professor of Opthalmology in the College of Physicians and Surgeons, Kansas City, Mo... 339

CLINICAL REPORTS.

Clinical Cases in Medical Wards of Mercy Hospital. By Professor Davis. From Notes by S........ 341

CORRESPONDENCE.

Letter from T. G. Williams, M.D., Watertown........ 346

PROCEEDINGS OF SOCIETIES.

Association of American Medical Editors. Third Annual Meeting........ 347

The Mutual Relations of the Medical Profession, its Press, and the Community. An Address delivered at the Annual Meeting of the Association of Medical Editors on May 1st, 1871, at San Francisco. By Horatio Robinson Storer, M.D., President of the Association.... 350

American Medical Association........ 360

President Stille's Opening Address..... 361

BOOK NOTICES.

Chemistry General, Medical, and Pharmaceutical, including the Chemistry of the U. S. Pharmacopœia. By John Attfield, Ph. D., F.C.S........ 376

Report of the Board of Health of the city of Chicago, for 1867, 1868, and 1869, and a Sanitary History of Chicago, from 1833 to 1870........ 377

Observations on the Physiological and Therapeutical Effects of Galvanization of the Sympathetic. By A. D. Rockwell, A.M., M.D., and Geo. M. Beard, A.M., M.D........ 377

Uterine Catarrh, frequently the cause of Sterility. New Treatment by H. E. Gantillon, M.D........ 377

Medical and Surgical Memoirs. By Joseph Jones, M.D., Prof. of Chemistry....... 377

EDITORIAL.

Correspondence of the Senior Editor.... 378

Illinois State Medical Society........ 383

Iowa State Medical Society........ 384

Carbolic Acid in Pruritus Cutaneous.... 384

Hiccough Cured by Chloral........ 385

Decline of Vaccination in Great Britain.. 385

Tetanus and Chloral Hydrate........ 385

Duration of Life........ 385

Deaths from Alcohol in Cities........ 385

Insanity........ 385

Intra-Uterine Injections........ 385

Wet Bandages in Fractures........ 386

The Anterior Splint........ 386

The Carbolic Acid Treatment in the London Hospitals........ 386

Iodoform Ointment........ 386

American Doctors........ 386

Cases of Poisoning from Chloral........ 386

